# Concurrent use of prescription drugs and herbal medicinal products in older adults: a systematic review protocol

**DOI:** 10.1186/s13643-016-0244-2

**Published:** 2016-04-21

**Authors:** Taofikat Agbabiaka, Barbara Wider, Leala Kay Watson, Claire Goodman

**Affiliations:** Centre for Research in Primary and Community Care (CRIPACC), University of Hertfordshire, Hatfield, UK; Patient Safety Domain, Nursing Directorate, NHS England, London, UK; Institute of Health Research, University of Exeter Medical School, Exeter, UK

**Keywords:** Concurrent use, Herbal medicinal product, Prescription drug, Prevalence, Herb-drug interaction, Adverse reaction, Safety, Elderly

## Abstract

**Background:**

There has been a global increase in the use of herbal medicinal products (HMPs). About a quarter of UK adults use HMPs, bought over the counter by self-prescription and often not disclosed to healthcare professionals. Potential herb-drug interaction is a clinical concern, with older people at greater risk because of co-morbidities and slower clearance of pharmacologically active compounds. While there is a good understanding of general herbal medicine use by older people, less is known about the extent and implications of concurrent use with prescription medicines. The aim of this systematic review is to assess the prevalence, patterns, safety issues and other factors associated with concurrent prescription and herbal medicines use among older adults.

**Methods/design:**

Systematic electronic searches of MEDLINE, PsychINFO, Excerpta Medica dataBASE (EMBASE), Cumulative Index to Nursing and Allied Health Literature (CINAHL), Allied and Complementary Medicine Database (AMED), Web of Science and Cochrane from inception till present for studies reporting the concurrent use of prescription medicines with HMPs in older adults (≥65 years). Lateral searching via related citation (PubMed) and checking reference lists of identified studies will be performed. Two reviewers will independently screen studies, extract data and appraise methodological quality using the Joanna Briggs Institute checklist for prevalence data and the Critical Appraisal Skills Programme (CASP) checklist. Qualitative and quantitative studies from all settings will be included. Non-empirical papers, in vitro experiments and animal studies will be excluded. Primary outcomes are prevalence and patterns of concurrent use, number and types of prescription and HMPs and adverse reactions reported. Secondary outcomes are disclosure of HMP use to healthcare professionals and cost of HMPs. A narrative synthesis of included studies will be performed to summarise the evidence.

**Discussion:**

This review will synthesise and critically appraise current knowledge on the concurrent use of drugs and HMPs by older adults and thus provide a better understanding of the issue. It will also identify any gaps in knowledge. By establishing safety issues associated with concurrent use, it will also inform strategies that can help practitioners to identify and manage older people at potential risk of herb-drug interactions.

**Systematic review registration:**

PROSPERO CRD42014009091

**Electronic supplementary material:**

The online version of this article (doi:10.1186/s13643-016-0244-2) contains supplementary material, which is available to authorized users.

## Background

The last two decades have witnessed a global increase in the use of herbal medicinal products (HMPs) [1]. Herbal medicinal product is described as “any medicinal product, exclusively containing as active ingredients one or more herbal substances or one or more herbal preparations, or one or more such herbal substances in combination with one or more such herbal preparations” [2]. High prevalence of HMPs use has been reported in the UK [3–5]. Herbal medicinal products are bought over the counter, mostly self-prescribed [5–7] and often not disclosed to healthcare professionals [8, 9]. Reasons for the increasing use of HMPs include dissatisfaction with the effectiveness of conventional drugs, concerns about adverse effects of conventional drugs and misconception that HMPs are ‘natural’ and therefore safer, even when or particularly when used with prescription drugs [7]. Other reasons for the increased use of HMPs include desire to contribute to the treatment process and to improve general health, cultural and personal beliefs and better consultation experiences with herbal medicine practitioners when compared to conventional healthcare professionals [10].

Interactions between conventional drugs and HMPs can lead to adverse drug reactions (ADR), some with serious consequences [11–14]. For example, ginkgo (*Ginkgo biloba*) when combined with anticoagulants can interfere with platelet functions. Although the majority of drug-drug interactions are known and preventable, little is known about the incidence and severity of HMP-drug interactions. The risk for ADRs increases with age [14, 15]. Considering that 16 % of the population of England and Wales are 65 years and older, this is an issue that needs closer attention [16]. Older people are more likely to live with multiple co-morbidities and experience medication-related problems because of delayed clearance of pharmacologically active compounds [17–19]. Twenty per cent of over 70s take five or more prescribed medications [20, 21]; the number of medications increases the risk of an adverse drug event [22, 23]. Increased hospitalisation and prolonged hospital stays due to ADRs are also major clinical and economic concerns for all healthcare systems [24]. While there is a considerable body of knowledge on the use of herbal medicines by different age groups and for different conditions, there is limited evidence on the concurrent use of herbals with conventional medicines especially among older adults [25]. We define concurrent use as taking one or more HMPs in the same time period with a prescription medicine.

### Aims

This systematic review aims to identify and synthesise the literature on prevalence, patterns, safety issues and other factors associated with concurrent prescription and HMP use in older adults.

### Review questions

The review will seek to answer the following questions:

- What is the prevalence and pattern of the concurrent use of prescription drugs and herbal medicinal products (HMPs) among older adults?
- What patient and clinical characteristics are associated with the concurrent use of prescription drugs and HMPs?
- What are the range of prescription drugs and HMPs most concurrently used by older adults?
- What safety issues and other factors are associated with concurrent prescription drug and HMPs use in older adults?

## Methods/design

### Protocol and registration

The methods for this systematic review were developed according to recommendations from the Preferred Reporting Items for Systematic Reviews and Meta-Analyses Protocols (PRISMA-P) statement [26]. A populated PRISMA-P checklist is available as an Additional file 1 to this protocol. The protocol has been registered in the International Prospective Register of Systematic Reviews: PROSPERO 2014: CRD42014009091.

### Eligibility criteria

We will include studies with participants who are ≥65 years, studies with a mean participant age of ≥65 years and studies from which standalone data for ≥65 years can be extracted. We will include studies assessing the concurrent use of HMPs or herbal dietary supplements with prescription medicines. Studies in which participants were on at least one prescription medicine and concurrently used one or more HMP will also be eligible.

### Type of studies

We will include the following study designs:

- Quantitative study design: quantitative studies which may include cross-sectional studies to answer review questions on prevalence, pattern of use, range of prescription and HMPs and patient and clinical characteristics associated with concurrent use among older adults
- Qualitative study design: qualitative studies on satisfaction or dissatisfaction with HMPs and disclosure to healthcare professionals, as well as cost and resources expended on HMPs by older adults
- Mixed method studies: studies which have incorporated both quantitative and qualitative strands in their design
- Case reports of adverse events from the concurrent use of prescription drugs and HMPs among those aged 65 years and older

Studies assessing Chinese herbal medicine, Ayurvedia, Kampo, Siddha, Unani, homoeopathic remedies, non-herbal dietary supplements and vitamins will be excluded as they often contain products other than plants. Combination products, i.e. products that contain herbal as well as non-herbal (vitamins, minerals) ingredients will be excluded. Editorials, letters, commentaries and papers reporting in vitro experiments and animal studies will be excluded.

The primary outcomes of interest include prevalence and pattern of concurrent use, names and number of concurrently used medicines, adverse reactions or potential herb-drug interactions reported. Secondary outcomes of interests are the disclosure of HMPs use to healthcare professionals, satisfaction with HMPs and cost of HMPs, where reported.

## Literature searches

We will search the following databases from inception to November 2015 using medical subject headings (MeSH) and text words related to ‘herbal medicine’, ‘prescription drugs’ and the scientific name and common names(s) of herbs most documented for concomitant use. Cumulative Index to Nursing and Allied Health Literature (CINAHL) via EBSCO, Cochrane Library, Excerpta Medica dataBASE (EMBASE), MEDLINE (via PubMed), the Allied and Complementary Medicine Database (AMED), PsychINFO, Web of Science and Google Scholar. No restrictions will be placed on language of publication. The search strategy is available as an Additional file 2 to this protocol.

We will also search reference lists of all identified studies and review articles for relevant references not identified by the electronic search. Key authors and experts in the field identified from reference list of included studies will be contacted for information about ongoing or unpublished studies. Duplicate studies will be recorded and discarded. We will also perform lateral searching using the related citation function in PubMed and cited by function in Google scholar to capture all relevant articles.

### Study selection process

Search results from all databases will be downloaded into Endnote and merged to remove duplicates. Two reviewers (Taofikat Agbabiaka (TA) and Barbara Wider (BW)) will scan all titles and abstracts for potential relevance. Any article for which there is uncertainty about relevance will be retained and the full text reviewed. Full text of all articles that may meet the eligibility criteria will be obtained and downloaded into Endnote.

Using a pre-designed eligibility checklist, two reviewers (TA and BW) will independently assess the full text of all obtained articles against the eligibility criteria and an eligibility code will be recorded. Studies that do not meet the inclusion criteria will be excluded and the reasons recorded. Any disagreements on eligibility will be resolved by discussion between two reviewers (TA and BW); if no consensus can be reached, a third reviewer (Claire Goodman (CG)) will be consulted.

### Data extraction

Using a piloted data extraction form, one reviewer (TA) will extract the data from included studies and a second reviewer (BW) will validate the extracted data. Key data to be extracted will include the following:

- Publication details: author (s), year of publication, title of paper, journal and country in which study was conducted and funding information
- Study design: study type, recruitment method and data collection method(s)
- Study aims, outcomes and measurement of outcomes.
- Participants: demographic and socio-economic characteristics, number surveyed, or interviewed and previous medical diagnosis
- Name/description and number of HMPs and name and number of prescription drug(s)
- Details of safety issues: adverse reactions and interactions reported
- Disclosure, satisfaction and cost/resource
- Study limitations: response bias, selection bias, etc.

Any disagreements will be resolved by a third reviewer (CG) or by achieving a consensus through discussion. Studies that do not meet the inclusion criteria will be excluded and the reasons recorded.

### Quality assessment

Two reviewers (TA and BW) will independently assess the methodological quality of each article that meets the inclusion criteria. Where appropriate, studies assessing prevalence will be assessed using the Joanna Briggs Institute (JBI) checklist for prevalence data [27]. Qualitative papers will be assessed for methodological rigour using the checklist developed by Critical Appraisal Skills Programme (CASP) [28].

### Data synthesis

We will adopt the EPPI-Centre three stages approach to mixed method research synthesis [29]. A first synthesis will aim to address prevalence, pattern of use and patient characteristics associated with the concurrent use of herbal medicinal products and prescription medicines. A second synthesis of qualitative research will address the fourth review question, i.e. safety issues and other factors such as disclosure, satisfaction and cost/resources. We do not anticipate many qualitative research papers; therefore, synthesis will depend on available data. We will use thematic synthesis to identify key themes, commonalities, and why HMPs are thought to be helpful or necessary [30]. Findings from both quantitative and qualitative stages will be summarised in a narrative account that addresses the review questions to provide a better understanding of concurrent medicine use among older adults.

Results of the search and study selection process will be presented in a flow chart [31]. Relevant data extracted from eligible studies will be presented as an evidence table. We will describe the study characteristics including details of study types, population characteristics and outcomes. We will provide a narrative synthesis of the summary of prevalence, patterns and safety issues from concurrent prescription and HMPs use in older adults. Where appropriate we will present data in tables and graphics. We will provide a detailed discussion of the limitations of the included studies and their implications on the findings. We will identify gaps in knowledge and provide suggestions for future research.

## Discussion

Failure to review and manage the concurrent use of prescription and herbal medicines in older adults is a considerable patient safety risk. Excessive and inappropriate medicine use is common among older adults [32, 33], and concurrent use with HMPs further complicates the problem.

This review is a significant addition to knowledge due to the paucity of evidence on the concurrent use of prescription drugs and HMPs in older adults. There is no up-to-date review on concurrent prescription and herbal medicine use among UK older adults; the only previous related study [34] is over a decade old. The review will provide healthcare professionals with up-to-date knowledge on the prevalence and factors associated with concurrent medication use among this population. It will also provide important baseline information for future intervention research.

### Dissemination

Findings from this systematic review will form part of TA’s doctoral thesis. The review will be prepared following PRISMA reporting standards for submission to a peer-reviewed journal with focus on care of older adults or drug safety and presented at conferences.

## Additional files

Additional file 1: Preferred Reporting Items for Systematic Review and Meta-Analysis Protocols (PRISMA-P) is an evidence-based minimum set of items for reporting in protocols of systematic reviews and meta-analyses. Additional file 2: Search strategy used for Medline detailing keywords, subject headings, search terms, search techniques and combination of search terms.

## Abbreviations

ADR: adverse drug reaction
AMED: Allied and Complementary Medicine Database
BW: Barbara Wider
CASP: Critical Appraisal Skills Programme
CG: Claire Goodman
CINAHL: Cumulative Index to Nursing and Allied Health Literature
EMBASE: Excerpta Medica dataBASE
HMP: herbal medicinal product
MeSH: medical subject heading
PRISMA-P: Preferred Reporting Items for Systematic Reviews and Meta-Analyses Protocols
PROSPERO: International Prospective Register of Systematic Reviews
TA: Taofikat Agbabiaka
