# Supplementary material for: Concurrent use of prescription drugs and herbal medicinal products in older adults: a systematic review protocol
Source: Syst Rev. 2016 Apr 21;5:65. doi: 10.1186/s13643-016-0244-2 (PMC4839096; doi:10.1186/s13643-016-0244-2)
Supplement: Additional file 2: — Search strategy used for Medline detailing keywords, subject headings, search terms, search techniques and combination of search terms. [file 13643_2016_244_MOESM2_ESM.docx]

**Additional file 2: Search strategy**

**Medline**

| 1 | herb*.ti,ab. |
| --- | --- |
| 2 | (plant* adj3 (caplet* or capsule* or compound* or cream* or decoction* or drug* or essence* or extract* or formul* or heal* or herb* or Infus* or juice* or medic* or mixture* or powder* or prepar* or prescri* or product or products or remed* or supplement* or tablet* or tea or teas or therap* or tincture* or tisane* or treatment*)).ti,ab. |
| 3 | (phytodrug* or phytomed* or phytopharmac* or phytother* or phytochemical*).ti,ab. |
| 4 | ((natural* or naturo*) adj3 (caplet* or capsule* or compound* or cream* or decoction* or drug* or essence* or extract* or formul* or herb* or Infus* or juice* or medic* or mixture* or powder* or prepar* or prescri* or product or products or remed* or supplement* or tablet* or tea or teas or therap* or tincture* or tisane* or treatment*)).ti,ab. |
| 5 | (botanical* adj3 (caplet* or capsule* or compound* or cream* or decoction* or drug* or essence* or extract* or formul* or heal* or herb* or Infus* or juice* or medic* or mixture* or powder* or prepar* or prescri* or product or products or remed* or supplement* or tablet* or tea or teas or therap* or tincture* or tisane* or treatment*)).ti,ab. |
| 6 | (Ethnobotan* or pharmacogno* or Ethnopharmaco* or ethnomedic*).ti,ab. |
| 7 | ("diet* supplement*" or "nutri* supplement*" or "food supplement*").ti,ab. |
| 8 | Pharmacognos*.ti,ab. |
| 9 | (traditional adj3 (caplet* or capsule* or compound* or cream* or decoction* or drug* or essence* or extract* or formul* or herb* or Infus* or juice* or medic* or mixture* or powder* or prepar* or prescri* or product or products or remed* or supplement* or tablet* or tea or teas or tincture* or tisane*)).ti,ab. |
| 10 | (folk adj3 (caplet* or capsule* or compound* or cream* or decoction* or drug* or essence* or extract* or formul* or herb* or Infus* or juice* or medic* or mixture* or powder* or prepar* or prescri* or product or products or remed* or supplement* or tablet* or tea or teas or tincture* or tisane*)).ti,ab. |
| 11 | (Aloe or aloes*).ti,ab. |
| 12 | ("black cohosh" or "actaea racemosa" or "Cimicifuga racemosa").ti,ab. |
| 13 | (Echinacea or "Coneflower").ti,ab. |
| 14 | ("Evening primrose" or "oenothera biennis").ti,ab. |
| 15 | (feverfew or "tanacetum parthenium" or "Chrysanthemum parthenium" or "Pyrethrum parthenium").ti,ab. |
| 16 | (garlic or "Allium sativum").ti,ab. |
| 17 | (ginger or "zingiber officinale").ti,ab. |
| 18 | (ginkgo or "fossil tree" or "maidenhair tree" or "Japanese silver apricot" or baiguo or "bai guo ye" or "kew tree" or yinhsing or "yin-hsing").ti,ab. |
| 19 | (ginseng or "Panax quinquefolius" or "Eleutherococcus senticosus").ti,ab. |
| 20 | (Grapefruit or "Citrus adj2 paradisi").ti,ab. |
| 21 | (Hawthorn* or Crataegus).ti,ab. |
| 22 | ("John's wort" or "Johns wort" or hypericum or "Klamath weed" or "goat weed").ti,ab. |
| 23 | (licorice or liquorice or glycyrrhzin or "Glycyrrhiza glabra" or "sweet root" or "gan zao").ti,ab. |
| 24 | ("saw palmetto" or serenoa).ti,ab. |
| 25 | (Soursop or "Annona muricata" or "durian blanda").ti,ab. |
| 26 | (valerian or "valeriana officinalis" or Valerianaceae).ti,ab. |
| 27 | (Mint or Mentha or peppermint or menthe or Spearmint).ti,ab. |
| 28 | exp Plants, Medicinal/ |
| 29 | exp ethnobotany/ or exp pharmacognosy/ |
| 30 | plant preparations/ or exp plant extracts/ |
| 31 | exp Ethnopharmacology/ |
| 32 | exp Dietary Supplements/ |
| 33 | Medicine, Traditional/ |
| 34 | exp Herb-Drug Interactions/ |
| 35 | exp Plant Exudates/ |
| 36 | materia medica/ or plant extracts/ |
| 37 | 1 or 2 or 3 or 4 or 5 or 6 or 7 or 8 or 9 or 10 or 11 or 12 or 13 or 14 or 15 or 16 or 17 or 18 or 19 or 20 or 21 or 22 or 23 or 24 or 25 or 26 or 27 or 28 or 29 or 30 or 31 or 32 or 33 or 34 or 35 or 36 |
| 38 | prescri*.ti,ab,kw. |
| 39 | ((conventional or synthetic) adj2 (drug* or medicin* or pharmaceut* or medicat*)).ti,ab,kw. |
| 40 | "drug therap*".ti,ab,kw. |
| 41 | ("over-the-counter" or "over the counter" or otc).ti,ab. |
| 42 | (("non-prescri*" or nonprescri*) adj2 (drug* or medicin* or pharmaceut* or medicat*)).ti,ab,kw. |
| 43 | ("behind-the-counter" or "behind the counter" or btc).ti,ab,kw. |
| 44 | exp Prescriptions/ |
| 45 | exp Drug Therapy/ |
| 46 | exp Prescription Drugs/ |
| 47 | exp Nonprescription Drugs/ |
| 48 | 38 or 39 or 40 or 41 or 42 or 43 or 44 or 45 or 46 or 47 |
| 49 | (elder* or old* or aged or geriatr* or Gerontol*).ti,ab,kw. |
| 50 | exp Aged/ |
| 51 | exp Geriatrics/ |
| 52 | 49 or 50 or 51 |
| 53 | 37 and 48 and 52 |
| 54 | Animals/ |
| 55 | Humans/ |
| 56 | 54 not (54 and 55) |
| 57 | 53 not 56 |
